# Validation of the Center for Neurologic Study Bulbar Function Scale–Chinese version in a population with amyotrophic lateral sclerosis

**DOI:** 10.1186/s13023-024-03255-1

**Published:** 2024-07-02

**Authors:** Shan Ye, Lu Chen, Davan Murphy, Jieying Wu, Hui Zhang, Hong Liu, Boliang Zou, Guanghao Hou, Nan Zhang, Tielun Yin, Richard A. Smith, Dongsheng Fan

**Affiliations:** 1 Department of Neurology, Peking University Third Hospital, 49 North Garden Road, Haidian District, Beijing, 100191 China; 2 Department of Neurology, Yan’an Hospital of Traditional Chinese Medicine, No. 26 Xuan Yuan Road, Bridge Ditch Street, Bao Ta District, Yan’an, Shaanxi Province 716000 China; 3 Center for Neurological Study in La Jolla, 7590 Fay Avenue, Suite 517, La Jolla, CA USA; 4 Key Laboratory for Neuroscience, National Health Commission/Ministry of Education, Peking University, 38 Xueyuan Road, Haidian District, Beijing, 100191 China; 5 Beijing Key Laboratory of Biomarker and Translational Research, Neurodegenerative Diseases, Beijing, 100191 China

**Keywords:** Amyotrophic lateral sclerosis, Center for neurologic study bulbar function scale, ALS functional rating scale–revised, Bulbar function

## Abstract

**Objective:**

The Center for Neurologic Study Bulbar Function Scale (CNS-BFS) was specifically designed as a self-reported measure of bulbar function. The purpose of this research was to validate the Chinese translation of the CNS-BFS_C_ as an effective measurement for the Chinese population with ALS.

**Methods:**

A total of 111 ALS patients were included in this study. The CNS-BFS_C_ score, three bulbar function items from the ALSFRS-R, and visual analog scale (VAS) score for speech, swallowing and salivation were assessed in the present study. Forty-six ALS patients were retested on the same scale 5–10 days after the first evaluation.

**Results:**

The CNS-BFS_C_ sialorrhea, speech and swallowing subscores were separately correlated with the VAS subscores (*p* < 0.001). The CNS-BFS_C_ total score and sialorrhea and speech scores were significantly correlated with the ALSFRS-R bulbar subscore (*p* < 0.001). The CNS-BFS_C_ total score and ALSFRS-R bulbar subscale score were highly predictive of a clinician diagnosis of impaired bulbar function (area under the receiver operating characteristic curve, 0.947 and 0.911, respectively; *p* < 0.001). A cutoff value for the CNS-BFS_C_ total score was selected by maximizing Youden’s index; this cutoff score was 33, with 86.4% sensitivity and 93.3% specificity. The CNS-BFS_C_ total score and the sialorrhea, speech and swallowing subscores had good–retest reliability (*p* > 0.05). The Cronbach’s α of the CNS-BFS_C_ was 0.972.

**Conclusion:**

The Chinese version of the CNS-BFS_C_ has acceptable efficacy and reliability for the assessment of bulbar dysfunction in ALS patients.

## Introduction

ALS is a serious neurodegenerative disease with a poor prognosis [1, 2]. Bulbar dysfunctions, such as dysarthria, dysphagia and sialorrhea, are major symptoms that may have an important relationship with quality of life [3].

In recent years, increasing attention has been given to the treatment of amyotrophic lateral sclerosis (ALS). In clinical research, bulbar function is often evaluated, especially in clinical trials, to improve bulbar function. Therefore, sensitive bulbar function evaluation tools are needed to assess functional changes.

Traditionally, the Amyotrophic Lateral Sclerosis Functional Rating Scale-Revised (ALSFRS-R) [4] has been utilized as a primary endpoint in clinical trials. In most settings, it is administered by a rater. In contrast, the Center for Neurologic Study Bulbar Function Scale (CNS-BFS) is a self-report scale [5]. The developers thought such a scale might be more useful for interrogating patients with bulbar dysfunction because speech and swallowing are highly nuanced functions. For example, while reliance on speech rate is a useful measure, it is simplistic when one considers the richness of verbal communication, which involves syntax, emotionality, attentiveness, facial expression, etc. In a recent treatment trial directed toward the enhancement of bulbar function, the CNS-BFS was superior to a medley of instruments that assess bulbar function: visual analog scales, timed measures of speech and swallowing, and the ALSFRS-R [5, 6].

The CNS-BFS was modeled on a previously developed and similar scale that is used to assess emotionality in neurologically impaired persons. In the case of the CNS-BFS, three domains of bulbar function were assessed using a self-rating format. Subjects rate each of these on a scale of 1 to 5. A value of 6 is assigned for each item in the speech domain in the case of people who are unable to speak. Scores ranged from 21 (subjects with normal function) to a high of 112. A sample question was provided prior to administering the test to familiarize patients with the scale’s methodology.

Evaluation scales are usually influenced by language and culture, especially for self-reports. Considering that no version of the CNS-BFS exists, we thought to remedy this. Accordingly, we aimed to validate a Chinese translation of the CNS-BFS_C,_ which conceivably should be an effective measurement for use in the Chinese population with ALS, as has been the case for its English version.

## Materials and methods

This study included 111 patients who were treated at Peking University Third Hospital in 2022 and met the revised El Escorial criteria for ALS diagnosis [7]. Patients who were illiterate and thus could not complete the evaluations were excluded. The clinical characteristics of the included patients are listed in Table 1. Forty-six ALS patients underwent retesting on a scale of 5–10 days after the first evaluation. This study was approved by the Research Ethics Committee of Peking University Third Hospital (IRB. No 00006761). In accordance with the Declaration of Helsinki, written informed consent was obtained from all participants before they were included. The consent procedure was approved by the ethics committee.

**Table 1:** Clinical characteristics of the ALS patients

Clinical characteristics	Value
Sex (male: female)	59:52
Age (years, mean ± SD)	53.93 ± 11.95
Education (years, mean ± SD)	11.64 ± 3.84
Duration of disease (months, mean ± SD)	24.07 ± 19.38
Bulbar dysfunction (yes: no)	66:45
Bulbar onset (yes: no)	26:85
Diagnostic level	
Definite	16/111
Probable	38/111
Laboratory-supported probable	33/111
Possible	24/111

The Chinese version of the CNS-BFS_C_ was generated by translating the English version with the permission of the original authors; the Chinese version was then translated back by another clinical neurologist who had never read the original English version. The back-translated English version was then sent to the author of the original manuscript for discussion and revision. Although there were language and cultural differences, we attempted to maintain consistency with the original document.

In addition to the Chinese version of the CNS-BFS_C_, three bulbar function items from the ALSFRS-R and the visual analog scale (VAS) for speech, swallowing and salivation were also assessed in the present study. The ALSFRS-R was administered by trained clinical doctors, and the CNS-BFS_C_ and VAS were self-administered by the patients. At least two experienced neurologists diagnosed impaired bulbar function on the basis of clinical symptoms and physical examinations. Clinical symptoms included patients and family members complaining of slurred speech, difficulty swallowing, coughing when drinking water and increased salivation. Physical examinations included dysarthria, tongue muscle atrophy and fibrillation, an increased or decreased gag reflex, and a positive mandibular and sucking reflex.

### Statistics

Multiple linear regression models were constructed for the total CNS-BFS_C_ score and total subscores. The factors included sex, age, years of education, diagnostic level, duration of disease, ALSFRS-R score, and VAS score. Separate receiver operating characteristic (ROC) curves were drawn for the CNS-BFS_C_ total score and the ALSFRS-R bulbar subscore. Cutoff values were selected by maximizing Youden’s index. Paired-sample t tests were used to evaluate the test–retest reliability of the CNS-BFS_C_ score, ALSFRS-R score and VAS score if the variables were normally distributed; otherwise, paired-sample Wilcoxon tests were used. Cronbach’s α was used to evaluate the internal consistency of the CNS-BFS_C_. A significance threshold of *p* < 0.05 was used for statistical inference. The statistical analysis was performed using SPSS 20.0 statistical software.

## Results

### Correlations between the CNS-BFS_C_ score and the ALSFRS-R score and VAS score

**Fig. 1:**
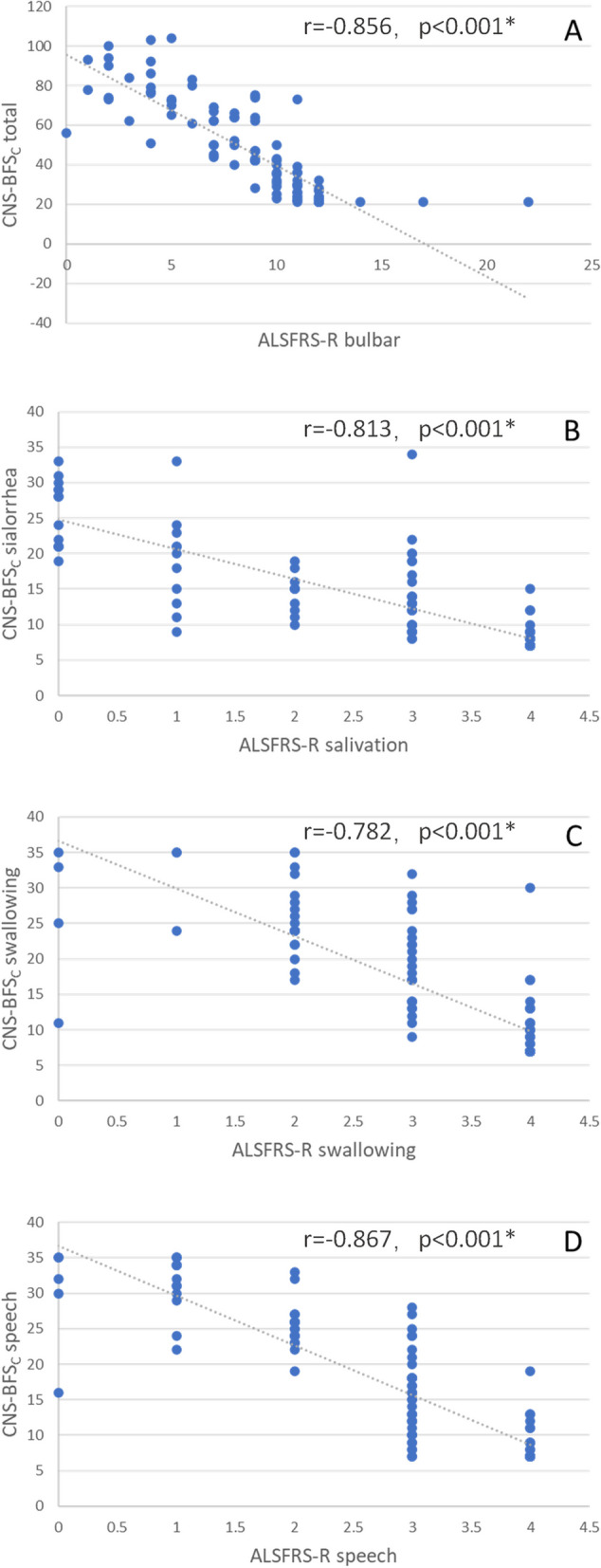
Correlations between CNS-BFS_C_ scores and ALSFRS-R scores. *p < 0.05

**Fig. 2:**
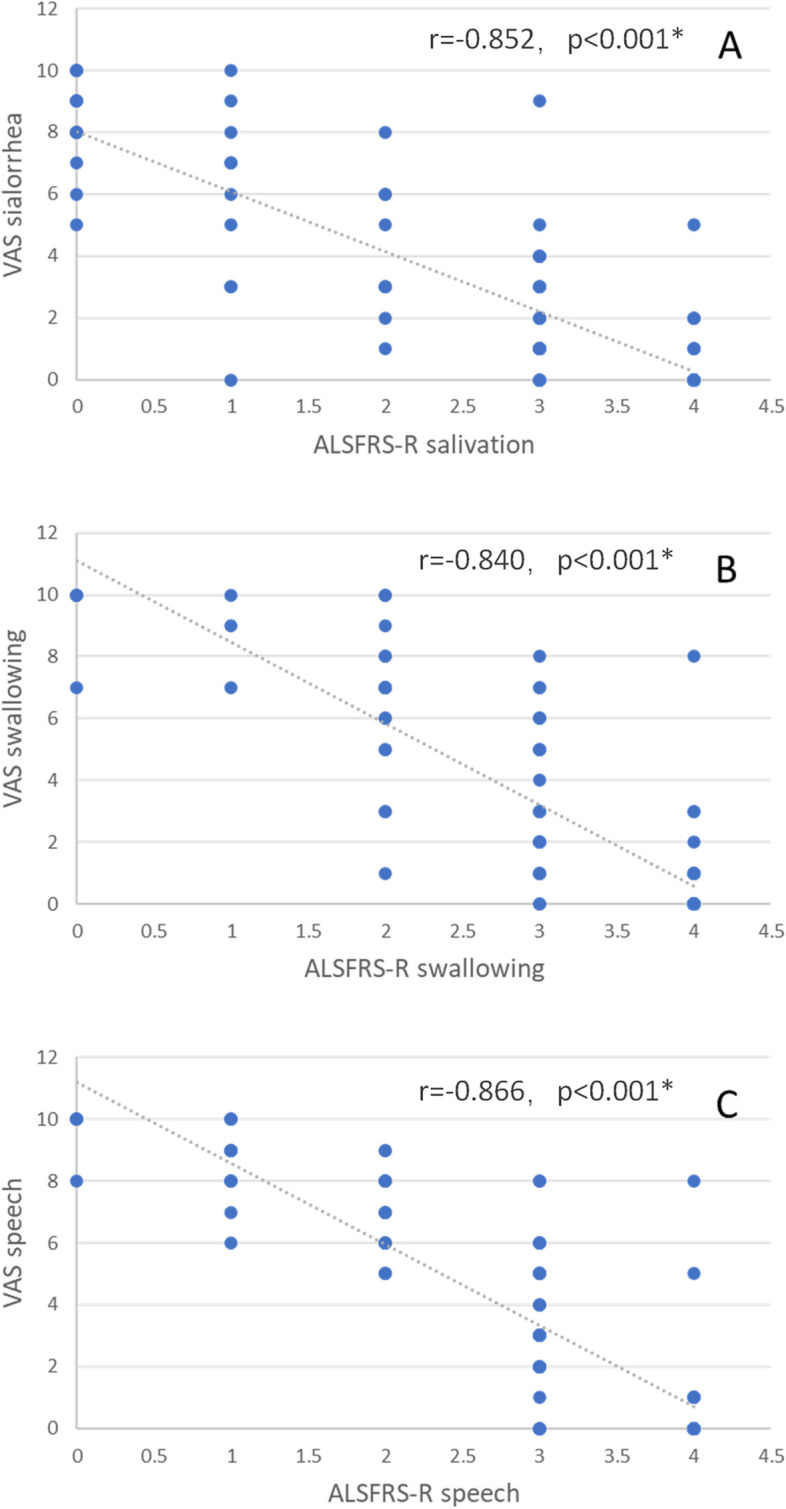
Correlations between CNS-BFS_C_ scores and VAS scores. *p < 0.05

**Fig. 3:**
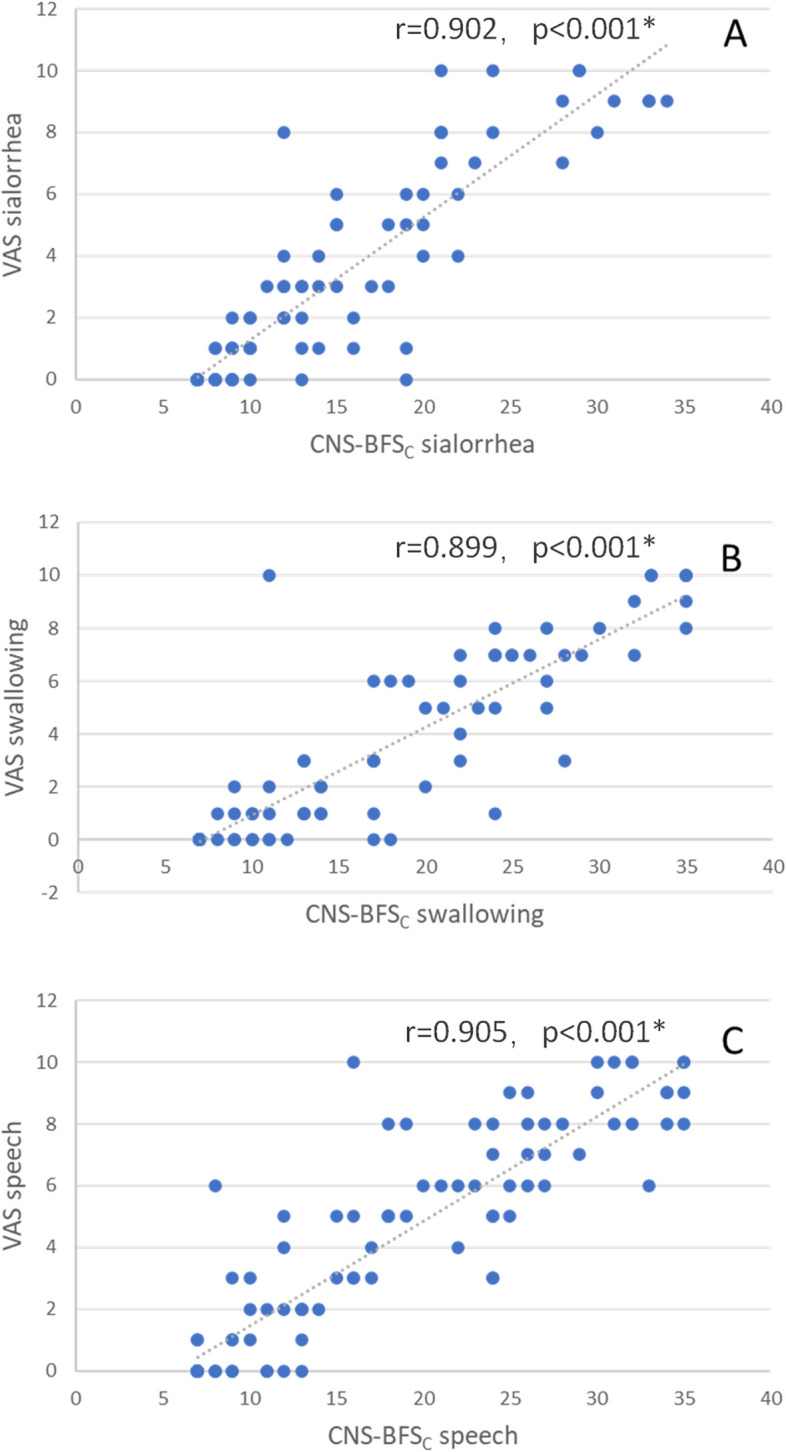
Correlations between the ALSFRS-R score and VAS score. *p < 0.05

The correlations between the CNS-BFS_C_ score and the ALSFRS-R score and VAS score are shown in Figs. 1, 2 and 3. Figure 1 suggests that the CNS-BFS_C_ total score may be linearly related to the ALSFRS-R bulbar subscore. Figure 3 shows that the CNS-BFS_C_ sialorrhea, speech, and swallowing scores may have strong linear relationships with the VAS sialorrhea, speech, and swallowing scores, respectively. Figure 2 shows that the ALSFRS-R bulbar subscores are related to the VAS score.

Multiple linear regression models were constructed for the total CNS-BFS_C_ score and total subscores (Table 2). The factors included sex, age, years of education, diagnostic level, duration of disease, ALSFRS-R score, and VAS score. The CNS-BFS_C_ sialorrhea, speech and swallowing subscores were correlated with the corresponding VAS subscores (*p* < 0.001). The CNS-BFS_C_ total score and sialorrhea and speech scores were significantly correlated with the ALSFRS-R bulbar subscore (*p* < 0.001), salivation score (*p* = 0.008, Bonferroni-corrected *p* = 0.032), and speech score (*p* < 0.001). After Bonferroni correction, disease duration had no significant relationship (*p* = 0.056) with the CNS-BFS_C_ sialorrhea score according to the linear regression model.

**Table 2:** Multiple linear regression of the CNS-BFS_C_ total score and subscores

CNS-BFS_C_ total			CNS-BFS_C_ sialorrhea			CNS-BFS_C_ speech			CNS-BFS_C_ swallowing		
Factors	t	*p*	Factors	t	*p*	Factors	t	*p*	Factors	t	*p*
Sex	-0.74	0.46	Sex	-0.42	0.673	Sex	-0.81	0.423	Sex	-1.15	0.255
Age	1.48	0.143	Age	1.96	0.053	Age	1.42	0.159	Age	0.76	0.452
Education	0.04	0.972	Education	-0.15	0.878	Education	0.40	0.689	Education	-0.41	0.686
Diagnostic level	0.16	0.875	Diagnostic level	-0.56	0.577	Diagnostic level	0.95	0.344	Diagnostic level	0.01	0.992
Duration of disease	-0.40	0.687	Duration of disease	-2.49	0.014^*^	Duration of disease	-1.29	0.200	Duration of disease	-0.53	0.600
ALSFRS-R bulbar subscore	-15.34	< 0.001^*^	ALSFRS-R salivation	-2.70	0.008^*^	ALSFRS-R speech	-3.99	< 0.001^*^	ALSFRS-R swallowing	-1.22	0.227
			VAS sialorrhea	9.57	< 0.001^*^	VAS speech	7.98	< 0.001^*^	VAS swallowing	10.08	< 0.001^*^

^*^p < *0.05*

### ROC analysis of the CNS-BFS_C_ total score and ALSFRS-R bulbar subscore

**Fig. 4:**
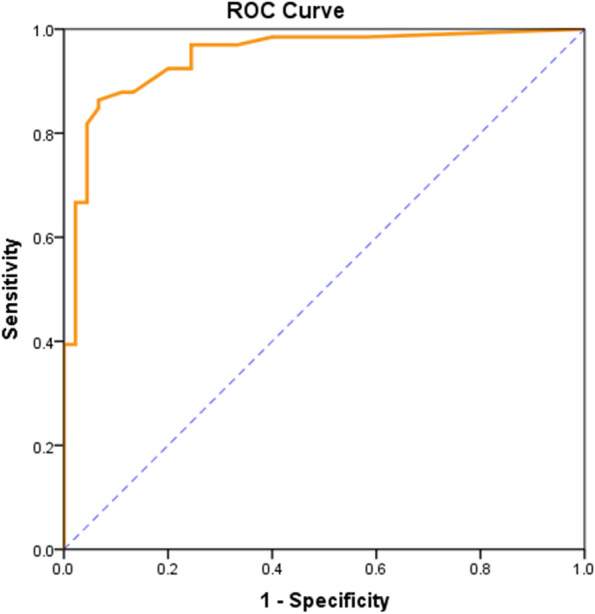
ROC curve of the CNS-BFS_C_ total score. Area under the ROC curve = 0.947; *p* < 0.001

**Fig. 5:**
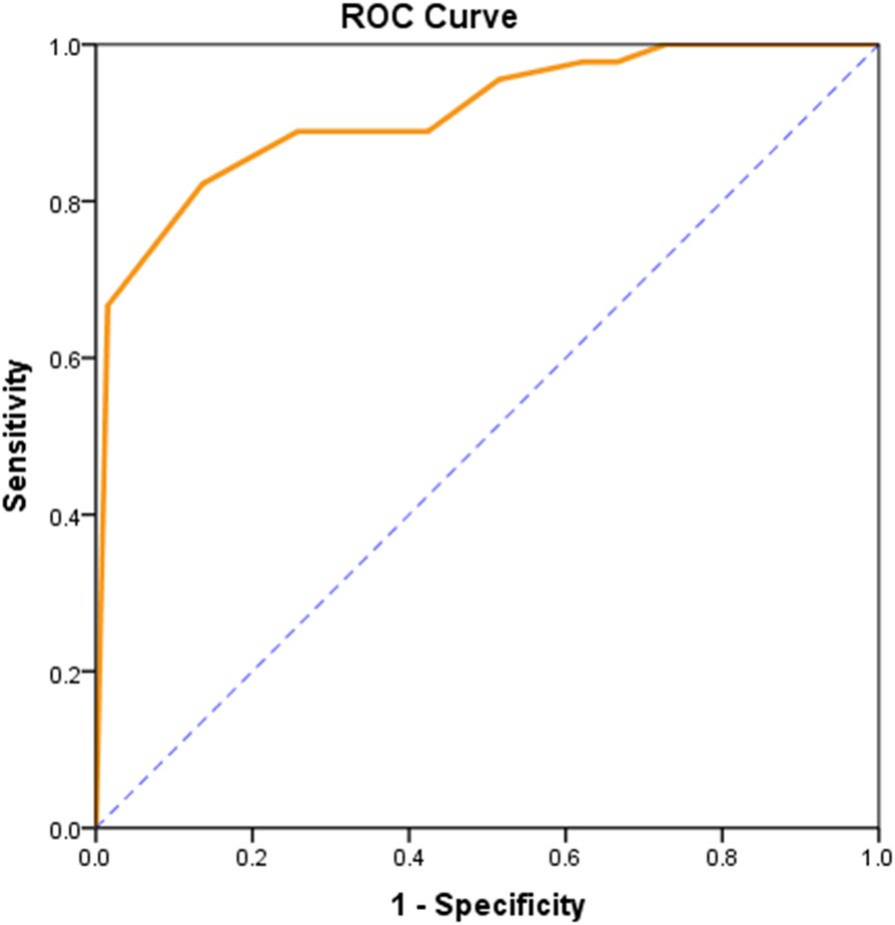
ROC curve of the ALSFRS-R subscore. Area under the ROC curve = 0.911; *p* < 0.001

The CNS-BFS_C_ total score and ALSFRS-R bulbar subscore were highly predictive of clinician diagnosis of impaired bulbar function (area under the ROC curve, 0.947 and 0.911, respectively; *p* < 0.001). Youden’s index was maximized at a CNS-BFS_C_ total score cutoff of 33; this cutoff achieved 86.4% sensitivity and 93.3% specificity (Figs. 4 and 5, Table 3).

**Table 3:** ROC curve features and cutoff values for the CNS-BFS_C_ total score and the ALSFRS-R bulbar subscore

Measures		CNS-BFS_C_ total score	ALSFRS-R bulbar subscore
ROC curve features	AUC	0.947	0.911
	Lower	0.905	0.854
	Upper	0.989	0.967
	*p* value	< 0.001^*^	< 0.001^*^
Cutoff defined by maximum Youden’s index	Value	> 33	≤ 10
	Accuracy	0.797	0.686
	Sensitivity	86.4%	82.2%
	Specificity	93.3%	86.4%

^*^p < *0.05*

### Test–retest reliability of the CNS-BFS_C_, ALSFRS-R and VAS scores

The CNS-BFS_C_ total score and sialorrhea, speech and swallowing subscores had good test–retest reliability (*p* > 0.05), while the ALSFRS-R salivation score did not (*p* = 0.038). The differences between the test and retest ALSFRS-R bulbar subscores showed trend-level significance (*p* = 0.062), and the same was true for the VAS swallowing scores (*p* = 0.07) (Table 4).

**Table 4:** Test–retest reliability of the CNS-BFS_C_, ALSFRS-R and VAS scores

	First test (mean ± SD)	Second test (mean ± SD)	t/z	*p*
CNS-BFS_C_ total	48.98 ± 23.44	48.57 ± 24.20	0.67	0.505
CNS-BFS_C_ sialorrhea	14.20 ± 7.34	14.24 ± 7.86	-0.13	0.901
CNS-BFS_C_ speech	18.80 ± 9.38	18.83 ± 9.43	-0.07	0.941
CNS-BFS_C_ swallowing	15.98 ± 8.56	15.50 ± 8.63	0.99	0.327
ALSFRS-R bulbar	7.80 ± 3.54	8.04 ± 3.64	-1.91	0.062
ALSFRS-R salivation	2.34 ± 1.46	2.61 ± 1.48	-2.14	0.038^*^
ALSFRS-R speech	2.43 ± 1.24	2.41 ± 1.26	0.37	0.710
ALSFRS-R swallowing	3.02 ± 1.18	3.02 ± 1.27	0.00	1.000
VAS sialorrhea	3.11 ± 3.41	3.09 ± 3.43	0.11	0.911
VAS speech	4.57 ± 3.66	4.72 ± 3.69	-1.23	0.227
VAS swallowing	2.93 ± 3.38	3.17 ± 3.70	-1.86	0.070

^*^p < *0.05*

### Internal consistency of the CNS-BFS_C_

The Cronbach’s α value of the CNS-BFS_C_ was 0.972, which suggested strong internal consistency.

## Discussion

This research was undertaken to determine the reliability of the Chinese version of the CNS-BFS_C_ in relation to its English version. First, in terms of comparability, the CNS-BFS_C_ total score was significantly correlated with the ALSFRS-R subscore. The ROC curves suggest that both the CNS-BFS_C_ total score and the ALSFRS-R bulbar subscore are highly predictive of impaired bulbar function as diagnosed by a clinician, although the CNS-BFS_C_ is somewhat better (0.947 vs. 0.911 AUC). The cutoff score of the original version of the CNS-BFS was 39 [5], while that of the CNS-BFS_C_ was 33. One possible reason is that the original version applied some objective indicators to evaluate bulbar dysfunction, such as speech rate and time to swallow liquids and solids; however, in the CNS-BFS_C,_ only symptoms and signs were considered.

The CNS-BFS_C_ subscores (for speech, swallowing, and sialorrhea) were strongly correlated with the ALSFRS-R and VAS subscores. Irrespective of how these scales are administered, the results suggest that the CNS-BFS_C_ compares favorably with both self-rated and clinician-rated scales. Since not all the ALS patients included in the study had bulbar dysfunction, it is reasonable that no relationship was found between disease duration and CNS-BFS_C_ score.

The ALSFRS-R is the most commonly used primary outcome measure in ALS clinical trials. However, the evaluation of bulbar function with that scale may lack sensitivity since there are only three items and the total possible score ranges only from 0 to 12 points. The CNS-BFS_C_ has 21 items, and the total possible score ranges from a low of 21 to a high of 112, which may make this scale more effective than the ALSFRS-R at detecting subtle changes during clinical trials.

The CNS-BFS_C_ total score and subscores all had good test–retest reliability, while the bulbar subscores of the ALSFRS-R did not. This might be because the bulbar subscores of the ALSFRS-R have much smaller range spans than those of the CNS-BFS_C_. Another reason might be the greater sensitivity of the ALSFRS-R to salivation, as this score was calculated by medical doctors. The internal consistency of the CNS-BFS_C_ was 0.972, which is a favorable outcome since a coefficient of 0.7–0.8 is considered good to excellent. Good reliability is essential for a scale because it enables comparisons across studies.

The CNS-BFS is a useful clinical tool during clinical trials, especially for evaluating bulbar dysfunction. It has been used in trials by Nuedexta [6, 8, 9]. Interestingly, in a previous study, when compared to those of the composite ALSFRS-R, the speech domains of both the CNS-BFS and the ALSFRS-R bulbar scale were sensitive measures of treatment efficacy. In contrast, the swallowing and salivation domains of the CNS-BFS were both responsive to treatment, whereas the swallowing and salivation questions on the ALSFRS-R were not [10]. Thus, CNS-BFS has been recommended for use in evaluating bulbar dysfunction [11]. In addition, the CNS-BFS can be applied as an important verification tool in the development of other objective, quantitative evaluation tools, such as the clinical bulbar assessment scale (CBAS) [12] and the amyotrophic lateral sclerosis-Bulbar dysfunction index (ALS-BDI) [13]. Like in the CNS-BFSC, the CNS-BFS_C_ also exhibited good sensitivity in our study. It might be widely used in the development of other objective evaluation tools, especially those based on deep studies of artificial intelligence, in the future [14–16].

Considering that Mandarin is one of the most prevalent spoken languages, the availability of a Chinese version of the CNS-BFS_C_ should be an important clinical and research tool for the evaluation and treatment of patients with ALS and likely kindred neurological disorders.

## Conclusions

The CNS-BFS-Chinese version has acceptable efficacy and reliability in the assessment of bulbar dysfunction in ALS patients. Although the CNS-BFS–Chinese version was validated in only the ALS population, it will likely be applicable to other diseases involving impairment of bulbar function.

## Data Availability

The data that support the findings of this study are available from the database of the Neurology Department, PUTH, Beijing, China. All the anonymized data within this article will be shared by request from any qualified investigator from the corresponding authors.
